# Differences in the chitinolytic activity of mammalian chitinases on soluble and insoluble substrates

**DOI:** 10.1002/pro.3822

**Published:** 2020-01-21

**Authors:** Benjamin A. Barad, Lin Liu, Roberto E. Diaz, Ralp Basilio, Steven J. Van Dyken, Richard M. Locksley, James S. Fraser

**Affiliations:** ^1^ Department of Bioengineering and Therapeutic Sciences University of California San Francisco California; ^2^ Biophysics Graduate Program University of California San Francisco California; ^3^ Tetrad Graduate Program University of California San Francisco California; ^4^ Science Education Partnership High School Intern Program, University of California San Francisco California; ^5^ Department of Pathology and Immunology Washington University School of Medicine in St. Louis St. Louis Missouri; ^6^ Department of Medicine University of California San Francisco California; ^7^ Department of Microbiology and Immunology University of California San Francisco California; ^8^ Howard Hughes Medical Institute San Francisco California

**Keywords:** chitin, directed evolution, enzymes, glycobiology

## Abstract

Chitin is an abundant polysaccharide used by many organisms for structural rigidity and water repulsion. As such, the insoluble crystalline structure of chitin poses significant challenges for enzymatic degradation. Acidic mammalian chitinase, a processive glycosyl hydrolase, is the primary enzyme involved in the degradation of environmental chitin in mammalian lungs. Mutations to acidic mammalian chitinase have been associated with asthma, and genetic deletion in mice increases morbidity and mortality with age. We initially set out to reverse this phenotype by engineering hyperactive acidic mammalian chitinase variants. Using a screening approach with commercial fluorogenic substrates, we identified mutations with consistent increases in activity. To determine whether the activity increases observed were consistent with more biologically relevant chitin substrates, we developed new assays to quantify chitinase activity with insoluble chitin, and identified a one‐pot fluorogenic assay that is sufficiently sensitive to quantify changes to activity due to the addition or removal of a carbohydrate‐binding domain. We show that the activity increases from our directed evolution screen were lost when insoluble substrates were used. In contrast, naturally occurring gain‐of‐function mutations gave similar results with oligomeric and insoluble substrates. We also show that activity differences between acidic mammalian chitinase and chitotriosidase are reduced with insoluble substrate, suggesting that previously reported activity differences with oligomeric substrates may have been driven by differential substrate specificity. These results highlight the need for assays against physiological substrates when engineering metabolic enzymes, and provide a new one‐pot assay that may prove to be broadly applicable to engineering glycosyl hydrolases.

## 1 INTRODUCTION

Chitin is a ubiquitous polysaccharide, comprised of ß‐1,4‐linked N‐acetylglucosamine, that is produced by fungi and arthropods for structural rigidity and water repulsion.^1^, ^2^ With notable exceptions,^3^ vertebrates generally do not produce chitin. However, mammals have a conserved machinery to recognize and degrade environmental chitin that is inhaled or ingested, and this machinery is tied to an innate immune response to chitin.^4^, ^5^

Chitin polymers assemble into water‐insoluble microcrystals, which have been observed in three different crystal forms, differentiated by the parallel or antiparallel orientation of neighboring chitin strands.^6^ Alpha‐chitin, the most common conformation, forms antiparallel sheets that intercalate the N‐acetyl groups of neighboring polymers and form tight hydrogen bonding networks.^7^ Strands of chitin must be extracted from this highly crystalline structure to be degraded, and the rate limiting step of catalysis has been observed to be the processive decrystallization of additional substrate from the bulk crystal.^8^, ^9^ This observation makes it particularly challenging to effectively associate degradation of short oligomeric analogues with true catalytic efficacy. The insolubility and recalcitrance of bulk chitin also makes it a particularly challenging substrate to quantify with high precision. Recently, several new methods have tackled this problem by using labelled chitin substrates with gel electrophoresis^10^, ^11^ as well as enzyme‐coupled assays to generate colorimetric signal from reducing ends.^12^ These methods have enabled new insights into chitinase behavior, but their signal‐to‐noise ratio and throughput limit the ability to separate total activity into binding and catalysis, as well as other components of polysaccharide catabolism such as substrate specificity and processivity.

The molecular mechanism of recognition of chitin and the signaling program generates in mammals is not well understood, but breakdown of inhaled chitin is accomplished by the secreted enzymes acidic mammalian chitinase (AMCase) and chitotriosidase, which are conserved across mammals.^4^ Both are two domain family‐18 glycosyl hydrolases consisting of a catalytic TIM‐barrel domain and a C‐terminal carbohydrate‐binding domain. In AMCase, the two domains are connected by a 25 residue glycine‐ and serine‐rich linker that is expected to be highly glycosylated, while chitotriosidase has a shorter, proline‐rich linker that has also been found to be glycosylated.^13^, ^14^, ^15^ The roles of the linker and the C‐terminal carbohydrate‐binding domain in processing chitin have not been quantified.

AMCase is upregulated in response to chitin insult and is secreted into the airway lumen, where it interacts with crystalline chitin and breaks down the substrate.^16^ Consistent with the reported role of AMCase in asthma, there are polymorphisms of human AMCase (hAMCase) that increase its activity and have been associated previously with asthma protection.^17^ A trio of mutations found far from the active site in the catalytic domain (15‐20 Å from active site inhibitor) in humans, N45D, D47N, and R61M, which change residues to the wild type identities of mouse AMCase (mAMCase), has been previously described to increase specific activity against model substrates.^18^ Of these mutations, prior work has identified the R61M mutation as causing the largest increase in total activity, as well as the largest decrease in mice with the reverse M61R mutation.^11^ The mechanism by which these mutations alter binding and catalysis remains unclear. AMCase deficient mice accumulate chitin in their lungs and develop tissue fibrosis as an aging phenotype; external addition of recombinant chitinase to the airway reduces this phenotype.^19^ This suggests that AMCase is predominantly responsible for clearance of chitin from airways, and further suggests that enhancing AMCase activity may reduce chitin airway levels.

In this study, we tried to evolve variants of AMCase that would have enhanced activity to test the hypothesis that enhanced clearance of chitin would reduce the potential for age‐related lung fibrosis. Our directed evolution approach was based on simple fluorogenic substrates. We found mutations that dramatically increase the activity of the enzyme by both improving binding and catalysis. We developed new approaches to quantifying bulk chitin degradation and discovered that these engineered mutations did not have the same effect with bulk substrates. We used these improved methods to assay the impact of the carbohydrate‐binding domain on activity and discover that it causes a minor *K*_*m*_ versus *k*_cat_ tradeoff but does not have a major effect on overall activity. We reverted the asthma‐protective mutants in the mouse background and find that the dominant effect is a *k*_cat_ decrease from the M61R mutation. We also compared the activity of mAMCase and chitotriosidase with different small oligomeric substrates and with bulk chitin. These results highlight the need for assays against more physiological substrates when engineering complex metabolic enzymes and provide a fluorigenic one‐pot reducing sugar assay that may be broadly applicable to engineering glycosyl hydrolases using realistic substrates.

## 2 RESULTS

### 2.1 *Engineering of hyperactive chitinases*

**Figure 1:**
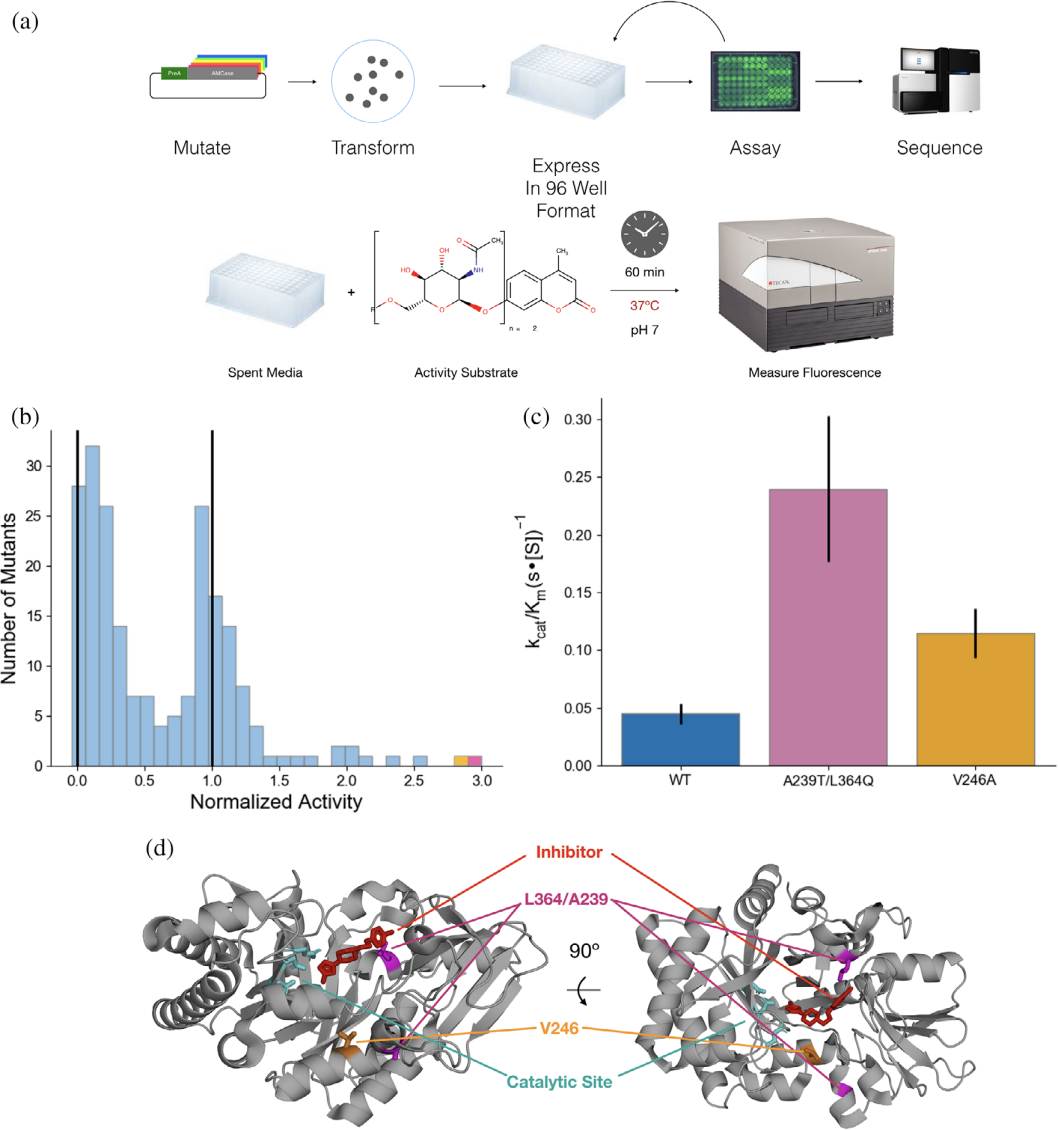
Engineering of hyperactive AMCase mutants. (a) Workflow for directed evolution of AMCase. Mutants of AMCase were generated via error‐prone PCR, then transformed and grown out from individual colonies in 96‐well blocks. After expression, activity was measured using the 4MU‐chitobioside substrate incubated with the expression media. (b) Distribution of activity for mutants with 1–3 mutations per construct. Vertical lines at 0 and 1 represent a catalytically dead negative control and a wild type positive control, respectively. The best two results are highlighted in purple and orange. (c) *k*_cat_/*K*_*m*_ of purified hyperactive mutants using the 4MU‐chitobioside assay (d) Structure of AMCase catalytic domain (PDB 3RM9) highlighting A239T/L364Q (pink) and V246A (orange). The active site catalytic network is highlighted in teal, and an inhibitor (5‐(4‐(2‐[4‐bromophenoxy]ethyl)piperazine‐1‐yl)‐1H‐1,2,4‐triazol‐3‐amine)^21^ that binds to the active site cleft is shown in red

Recent efforts have identified recombinant chitinase as a potential direct therapy to ameliorate inflammatory lung symptoms that arise when native chitinase activity is compromised.^19^ To investigate whether we could improve the activity of mouse AMCase, we used error‐prone PCR to generate libraries of mAMCase mutants (Figure 1a). Our recombinant expression approach, utilizing periplasmic secretion as described previously, also yields enzyme secreted into the media.^20^ We therefore assayed, in 96 well format, the ability of the spent media of individual mutants after protein expression to cleave 4MU‐chitobioside (Figure 1a). Comparing these results to both wild‐type and engineered catalytically dead mutants, we found that while most mutations resulted in either total loss of protein activity or similar activity to wild‐type, a small number of mutants were much more active than the wild‐type (Figure 1b). Because these assays were done directly on spent media, the measured activity for each well reports on the combination of the specific activity of the enzyme, expression level, and secretion efficiency. To determine whether our results represented improvements in activity, we isolated and purified the two most active mutants: A239T/L364Q (Figure 1d, pink) was the most active mutant identified, with a 5‐fold improvement in activity, and V246A (Figure 1d, orange), which showed a 2‐fold improvement in activity.

After purification, we measured the specific activity of the assay using a one‐pot continuous‐read fluorescent assay based on the previously developed enzyme‐coupled assay^12^ and replicated the improvements observed in the unpurified screening format (Figure 1c). Both mutants improved significantly in *k*_cat_, while the A239T/L364Q had a nonsignificant improvement in *K*_*m*_ (Table 1). Structurally, the V246A mutation may have a second‐shell interaction stabilizing the active conformation, while L364Q is positioned at the binding site for chitin and may directly improve chitin hydrolysis (Figure 1d).

**Table 1:** Measured rate constants for engineered mutants using 4MU‐chitobioside assay

	*k*_cat_	Fold change (*p*‐value)	*K*_*m*_	Fold change (*p*‐value)
WT	1.5 ± 0.3	N/A	33 ± 12	N/A
A239T/L364Q	4.5 ± 1.2	3.0 (*p* = .014)	19 ± 9	0.58 (*p* = .18)
V246A	3.6 ± 0.7	1.4 (p = .009)	32 ± 12	0.97 (*p* = .92)

*Note*: *k*_cat_ values are reported in units of 1/s. *K*_*m*_ values are reported in units of mM for 4MU‐chitobioside. Fold changes are relative to wild‐type enzyme.

### 2.2 *Comparison of the activity of the catalytic domain of AMCase to the full length enzyme with new approaches*

An alternative hypothesis for the increased activity is that the engineered variants have high specificity for the fluorophore or a smaller oligomer. This motivated us to develop new assays on larger and more complex chitin material. As a first control, we first assessed the contribution of the catalytic and carbohydrate‐binding domain of AMCase. Due to its small oligomeric size, hydrolysis of the 4MU substrate is likely to be driven only by local interactions in the catalytic domain and the presence of the carbohydrate‐binding domain should not affect the reaction rate. In contrast, the carbohydrate‐binding domain has been hypothesized to play a role in binding crystalline chitin.^22^, ^23^

**Figure 2:**
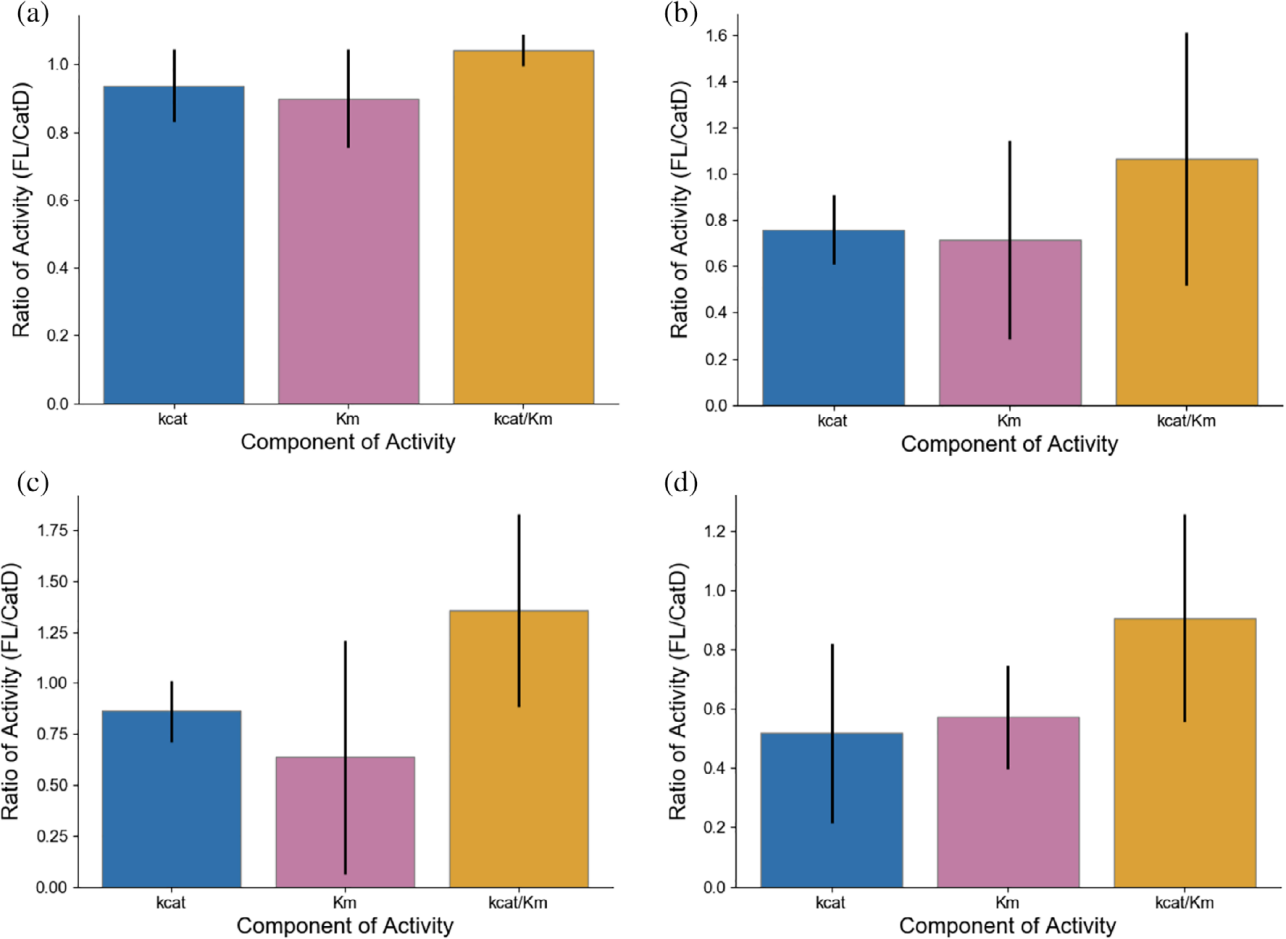
Activity comparisons of AMCase catalytic domain and full length enzyme. Difference in *k*_cat_, *K*_*m*_, and *k*_cat_/*K*_*m*_ of AMCase catalytic domain and full length enzyme generated via (a) 4MU‐chitobioside assay, (b) colloidal chitin clearance assay, (c) reducing sugar generation assay quantified with potassium ferricyanide, (d) chitooligosaccharide oxidase coupled peroxidase assay. Error bars denote propagated *SD* of fit (accounting for covariance)

We expressed and purified the isolated catalytic domain of AMCase, as well as the full length enzyme, using an *E. coli* periplasmic expression approach.^20^ We first measured the ability of the enzyme to catalyze the breakdown of 4‐methylumbelliferone (4MU) conjugated chitobioside, using a continuous read approach at pH 7.0. The activities of the two constructs were indistinguishable, either in binding or catalysis (Figure 2a, Table 2, *p* = .3).

**Table 2:** Calculated rate constants of AMCase catalytic domain and full length enzyme

	Catalytic domain		Full length enzyme	
	*k*_cat_	*K*_*m*_	*k*_cat_	*K*_*m*_
4MU‐chitobioside	1.12 ± 0.09	28 ± 3	1.05 ± 0.07	25 ± 2
Colloidal clearance	0.00140 ± 0.00008	0.09 ± 0.03	0.00106 ± 0.00002	0.07 ± 0.02
Ferricyanide	0.454 ± 0.042	0.046 ± 0.02	0.39 ± 0.02	0.029 ± 0.007
ChitO	0.9 ± 0.1	0.033 ± 0.006	0.54 ± 0.08	0.017 ± 0.005

*Note*: *k*_cat_ values are reported in units of 1/s. *K*_*m*_ values are reported in units of mM for 4MU assays and %w/v for colloidal clearance, ferricyanide, and chitO assays.

**Table 3:** Calculated rate constants of engineered mutants with *k*_cat_ values are reported in units of 1/s

	*k*_cat_	Fold change (p value)	*K*_m_	Fold change (p value)
WT	0.78 ± 0.05	N/A	0.030 ± 0.002	N/A
A239T/L364Q	N.D.	N/A	N.D.	N/A
V246A	0.80 ± 0.07	1.03 (*p* = 0.7)	0.035 ± 0.004	1.17 (p = 0.1)

*Note*: *K*_m_ values are reported in units % w/v. Fold changes are relative to wild type enzyme.

We next tested different methods of quantifying hydrolysis of insoluble chitin. We used colloidal chitin substrates, which are more uniform in size and shape and to have reduced settling times compared to other substrates such as shrimp shell chitin. We first attempted to measure colloidal chitin hydrolysis by the disappearance of scattering by solid substrate as it is converted into small oligomeric products. We could not distinguish a statistically significant difference between the two variants with this approach, which was likely limited by the relatively small dynamic range and large amount of enzyme required to produce a measurable change in scattering (Figure 2b, Table 2, *p* = .8). Each hydrolysis event only minimally alters the scattering of chitin crystals, and many cuts are likely necessary to solubilize crystals.

We next attempted to quantify the production of soluble reducing ends, which we hypothesized would more sensitively report individual catalytic events. The first method we used to assay production of soluble reducing ends was a ferricyanide reduction assay^24^: after incubating colloidal chitin with AMCase at 37°C for up to 18 hr, we quenched the reaction and quantified the nonenzymatic reaction of soluble reducing sugars with potassium ferricyanide, read out by the disappearance of absorbance at 420 nm. With this assay, we were not able to identify a significant difference in total activity but were able to identify that the inclusion of the carbohydrate‐binding domain created a small improvement in *K*_*m*_ that was offset by a reduction in the *k*_cat_ of AMCase (Figure 2c, Table 2, *p* = .2). This tradeoff did not result in a large difference in activity. Moreover, the endpoint‐based requirements of the assay and of the dynamic range available in measuring reduction in absorbance were limiting. We next developed a new assay based on previous work using chitooligosaccharide oxidase (chitO) in combination with horseradish peroxidase to generate signal specifically from the production of chitin reducing ends.^12^ To convert this assay from endpoint to continuous readout, we took advantage of fluorogenic substrates for horseradish peroxidase and carefully washed the colloidal chitin to enable signal measurement without removal of the insoluble component. This gain‐of‐signal fluorescent assay had much improved signal‐to‐noise and sensitivity, and improved quantification of the kinetic parameters of chitinase activity. Using this assay, we were able to more confidently determine the tradeoff between improved binding (*p* = .02) and loss of maximal catalytic activity (*p* = .009) with the inclusion of the carbohydrate‐binding domain, which resulted in no significant change in total activity (Figure 2d, Table 2, *p* = .8).

### 2.3 *Small substrates can be misleading for engineered chitinases*

**Figure 3:**
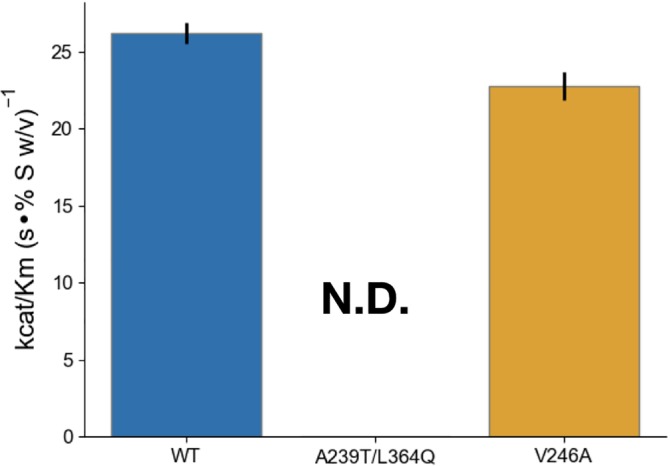
Engineered mutant activity with the novel chitooligosaccharide oxidase assay. Difference in *k*_cat_/*K*_*m*_ of purified hyperactive mutants using the 4MU‐chitobioside assay. *k*_cat_ values are reported in units of 1/s. *K*_*m*_ values are reported in units % w/v. Error bars denote propagated *SD* of fit (accounting for covariance). The A239T/L364Q mutant had too little total activity to measure *k*_cat_ or *K*_*m*_

Having this new assay in hand, we tested whether the activity increases observed with the 4MU‐chitobioside mutant resulted in similar improvements to degradation of bulk chitin. Using purified protein, we measured the activity of the mutants to degrade colloidal chitin using the enzyme‐coupled chitO assay, and discovered that the A239T/L364Q mutant had lost all measurable activity, while the V246A mutant was not statistically significantly more active than the wild type (Figure 3, Table 3). The loss of activity of the double mutant suggests that the improvements were driven by the L364Q mutation interacting with the 4MU fluorophore, which can be rationalized structurally (Figure 1d). The stark difference in results between the results with the 4MU and chitO assay underscores the need for assays of catabolism of bulk chitin substrate, even during the initial stages of screening.

### 2.4 *Effects of human asthma‐associated mutants in the mouse context*

**Figure 4:**
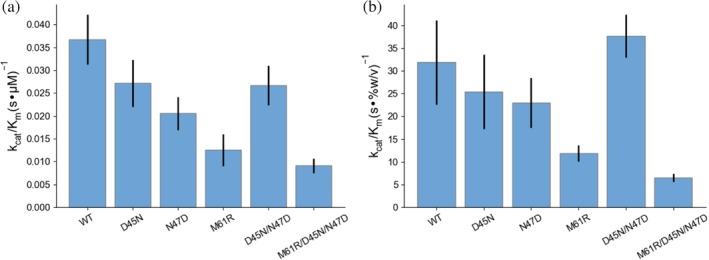
Comparison of activity of AMCase asthma‐associated mutants. Measurement of *k*_cat_/*K*_*m*_ for reversed asthma‐associated mutants in the mouse background using the (a) 4MU‐chitobioside and (b) chitO assays. Error bars denote propagated *SD* of fit (accounting for covariance)

Motivated by the result on the engineered mutations, we wanted to test naturally occurring mutations that have previously been shown to have different activities using the 4MU assay. We focused on a trio of mutations in AMCase in humans, N45D, D47N, and R61M, that confer significantly increased activity to AMCase.^11^, ^18^ In all three cases, the identity of the mutated residues becomes the same as the identity of the residues of the mouse wild‐type protein. To better understand the mutational landscape between the mouse and human enzymes, which have 81% sequence identity and differ by 92 total polymorphisms, we made the reverse mutations in the mouse background to quantify their effect on activity using both 4MU‐chitobioside and bulk chitin. First we measured the activity of the mutations using 4MU‐chitobioside, which showed that the mouse wild‐type residues were more active than the human wild‐type residues. The activity difference between wild‐type and the M61R mutant was caused by a decrease in *k*_cat_ and a small increase in *K*_*m*_ (Figure 4a, Table 4, *p* = .03). Smaller effects were observed for the individual D45N and N47D mutations, but the effects were reversed by the charge‐swapped D45N/N47D construct. The full triple mutant was the least active (*p* = .001). These results show strong alignment with previous results in the human background^11^ and suggest that the different residue identities have very similar effects in the mouse and human backgrounds. To understand whether these effects observed with the oligomeric substrate are relevant to enzyme activity on bulk chitin, we assayed the activity of humanizing mutations in mAMCase using the enzyme‐coupled chitO assay. The results were similar to those using the 4MU substrate, with the largest effect of any individual mutation and the majority of the effect of the triple mutation contributed by the M61R mutant (Figure 4b, Table 4, *p* = .02). The effects of the D45N and N47D mutations were less pronounced in the chitO assay, while the M61R mutation had a similar effect on both *K*_*m*_ and *k*_cat_.

**Table 4:** Calculated rate constants for AMCase and chitotriosidase *k*_cat_ values are reported in units of 1/s

	4MU‐chitobioside		chitO	
	*k*_cat_	*K*_*m*_	*k*_cat_	*K*_*m*_
WT	1.1 ± 0.1	30 ± 3	1.0 ± 0.2	0.032 ± 0.008
D45N	0.94 ± 0.07	35 ± 6	0.8 ± 0.1	0.032 ± 0.008
N47D	0.71 ± 0.05	35 ± 6	0.74 ± 0.03	0.032 ± 0.008
M61R	0.8 ± 0.2	60 ± 12	0.48 ± 0.05	0.041 ± 0.005
D45N/N47D	0.98 ± 0.09	37 ± 5	1.02 ± 0.04	0.027 ± 0.003
D45N/N47D/M61R	0.46 ± 0.05	50 ± 7	0.31 ± 0.04	0.047 ± 0.006

*Note*: *K*_m_ values are reported in units of mM for 4MU‐chitobioside and % w/v for chitO assays.

### 2.5 *Comparison of acidic mammalian chitinase and chitotriosidase*

Next we wanted to compare AMCase to the other major human chitinase, Chitotriosidase. Both enzymes are expressed in lungs, but only acidic mammalian chitinase is strongly overexpressed in response to chitin insult.^25^ Previous reports using the 4MU assay have indicated activity differences and no synergistic effects,^26^ but this result is convolved with the substrate specificity of dimer and trimer chitin oligomers. Whether AMCase and chitotriosidase have similar activity on crystalline substrates has not been previously examined.

**Figure 5:**
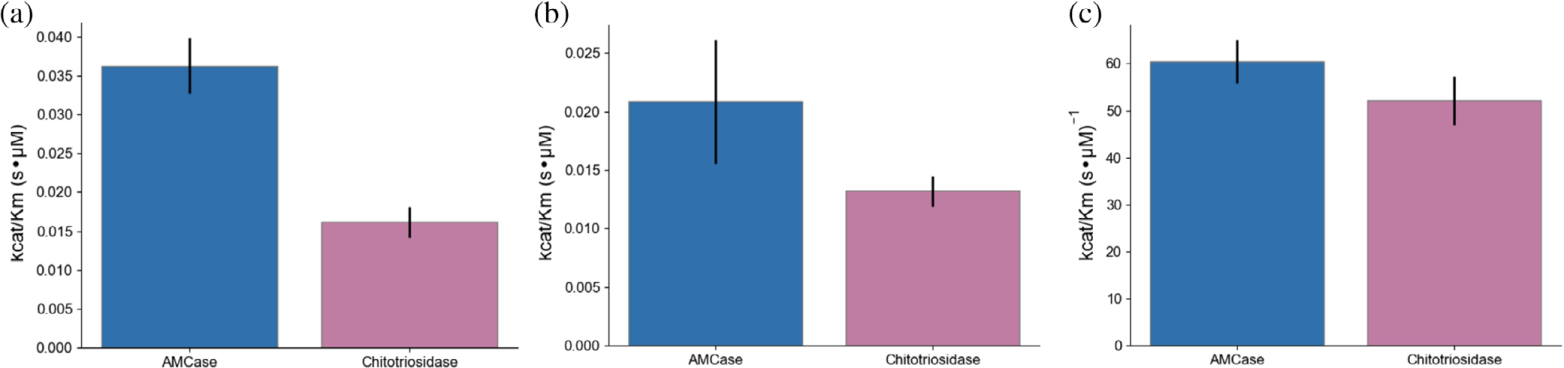
Comparison of AMCase and Chitotriosidase. Differences in *k*_cat_/*K*_*m*_ between AMCase (blue) and chitotriosidase (magenta) using (a) 4MU‐chitobioside, (b) 4MU‐chitotrioside, (c) chitooligosaccharide oxidase coupled peroxidase assay. Error bars denote propagated *SD* of fit (accounting for covariance)

We sought to understand how binding, substrate specificity, and hydrolytic activity differed between the two enzymes. We investigated substrate specificity by comparing the ability of each enzyme to cleave the terminal glycosidic linkage on 4MU‐chitobioside and 4MU‐chitotrioside, representing hydrolysis in different substrate binding poses to generate chitobiose versus chitotriose as a substrate. When assayed the 4MU‐chitobioside substrate, AMCase had more than double the activity of chitotriosidase, driven by a significant difference in *K*_*m*_ (Figure 5a, Table 5, *p* = .01). In contrast, the 4MU‐chitotrioside substrate led to tighter binding for both AMCase and chitotriosidase, but the difference was much larger with chitotriosidase, leading to a smaller gap in activity between the two enzymes (Figure 5b, Table 5). The reduction in observed *k*_cat_ for both enzymes was likely driven by the alternative, nonfluorogenic reaction trajectory in which the 4MU‐chitotrioside is cleaved into chitobioside and 4MU‐bound N‐acetylglucosamine, leading to a systematic underestimate of *k*_cat_. The differences in the *K*_*m*_ suggests that chitotriosidase benefits more from the extended binding interactions available with the larger 4MU‐chitotrioside substrate. We next assayed the differences in activity with a bulk substrate using the chitO‐coupled assay. The difference in activity was much smaller in this assay, with the majority of the activity difference being driven by *k*_cat_ differences (Figure 5c, Table 5, *p* = .006). These results further confirm that much of the apparent activity differences between AMCase and chitotriosidase are due to differential substrate specificity, as had been previously described,^26^ and suggest that much of this difference can be attributed to differential binding efficiency for short chitin oligomers.

**Table 5:** Calculated rate constants for AMCase and Chitotriosidase *k*_cat_ values are reported in units of 1/s

	AMCase		Chitotriosidase	
	*k*_cat_	*K*_*m*_	*k*_cat_	*K*_*m*_
4MU‐chitobioside	1.02 ± 0.05	28 ± 4	1.1 ± 0.1	68 ± 15
4MU‐chitotrioside	0.5 ± 0.1	24 ± 11	0.33 ± 0.03	25 ± 4
ChitO	1.06 + 0.06	0.018 ± 0.002	0.82 ± 0.05	0.016 ± 0.003

*Note*: *K*_m_ values are reported in units of mM for 4MU assays and % w/v for chitO assays.

## 3 DISCUSSION

Broadly, these results demonstrate the value of quantifying chitinase kinetics with bulk substrates with the same care used with model substrates (fluorogenic oligomers). Our results suggest that the effectiveness and sensitivity of the one‐pot chitooligosaccharide oxidase coupled assay makes it an ideal approach for monitoring chitinase activity. While in some cases, the results of the activity assays closely resembled the 4MU‐chitobioside assays, in others, the activities were tremendously different, underscoring the need for quantitative measures of bulk chitin catabolism. This proved to be particularly true for studies of the effects of multiple domains, which necessarily cannot bind the same short oligomer the same way they could a chitin crystal, as well as for engineered variants, in which screening with short fluorogenic substrates led to artifacts that may be related to fluorophore binding. The sensitivity and throughput available with the chitO‐coupled assay enables more precise and quantitative measurements of bulk chitin catabolism than was previously available, and we expect that this technique will be effective for deconstructing different aspects of enzyme activity.

In contrast to the majority of cases, which had reasonable agreement between the bulk experiments and the small oligomers, our efforts to engineer hyperactive chitinases were limited by the use of the 4MU‐chitobioside substrate as a screening tool. Our best mutants from screening had significant increases in activity, but once the purified mutants were assayed by the chitO assay, the improvements were not present. In the case of the A239T/L364Q mutant, there was no quantifiable activity with bulk substrate. The classic maxim is that “in protein engineering you get what you screen for”, and in this case that was maximizing binding efficiency for the 4‐methylumbelliferone fluorophore and the chitin dimer. The result underscores the need in the future for utilizing frequent counter‐screening with bulk chitin when performing selection experiments for chitin processing and matches well with previous results in engineering cellulases, which showed that screening with synthetic substrates had significant pitfalls compared to using insoluble substrates.^27^ One challenge to accomplishing this is that, while the chitO assay is more sensitive and high throughput than previous techniques, it is sensitive to free sugars and other components of the media that limits its utility for direct screening. With small scale purification, we may in the future be able to directly screen activity of mutants using the chitO method. In combination with recent advances in guiding small library directed evolution with machine learning,^28^ we may be able to effectively use this approach to find hyperactivating mutants without the requirement of using chitobioside substrates.

With the exception of the engineered mutants, the kinetic parameters measured with the 4MU and bulk chitin assays were well aligned, with *k*_cat_ values that were remarkably similar, suggesting that the 4MU assay effectively captures the chemical step of hydrolysis, and *K*_*m*_ values that were on the order of 30 μM for the 4MU‐chitobioside and 0.03% w/v for the bulk chitin assay. Under the approximation of infinite polymer length, there is one binding site per N‐acetylglucosamine unit. Each chitin monomer unit has a molecular mass of 203.21 g/mol, so 0.03% w/v or 0.3 g/L would correspond to approximately 1.5 mM, 50 times greater than the *K*_*m*_ observed for the small oligomeric substrates. We hypothesize that the higher effective *K*_*m*_ reports on the relative crystallinity of the chitin, with a small proportion of theoretical substrate binding sites being accessible to the enzyme. In the future, it may be possible to alter this crystallinity, using partial deacetylation, coapplication of chitin‐binding enzymes that might loosen the crystalline geometry, or physical milling to alter the surface area to volume ratio.

Using the new bulk activity measurements, we were able to discern a tradeoff between *k*_cat_ and *K*_*m*_ with the addition of the carbohydrate‐binding domain of AMCase, as *K*_*m*_ improved from 0.0333% ± 0.0056% to 0.0172% ± 0.0049% chitin w/v (*p* = .02), while *k*_cat_ decreased from 0.944 ± 0.111 1/s to 0.540 ± 0.083 1/s (*p* = .007). While the improved binding with the addition of additional binding sites for chitin is unsurprising, the difference in the *k*_cat_ is less clear. Previous work has suggested that, for some chitinases, the rate limiting step in bulk catalysis is processivity.^9^ This result supports that hypothesis for AMCase as well, since the additional binding motif may inhibit the ability of the catalytic domain to effectively slide to new binding sites. If AMCase processivity proves to be rate limiting, given the closely matched *k*_cat_ for 4MU‐chitobioside, with which processivity is not possible, and bulk chitin, it suggests that the rates of catalysis and processivity may be very similar in the mouse enzyme. This may be a result of selection optimizing the overall rate of the enzyme or the relative size of products generated by the enzyme. For example, larger oligomers could be produced if decrystallization and sliding were much faster than the rate of hydrolysis. These larger oligomers may be the relevant molecules sensed by the mammalian immune system, as seen in plants.^29^ The carbohydrate‐binding domain may further impact other aspects of catalysis, such as selecting specific chitin local morphology, binding chitin in the correct orientation, modulating processivity, or releasing when strands of chitin become too short to further process. Additionally, the assayed constructs lack posttranslational modifications. Acidic mammalian chitinase is predicted to have multiple O‐linked glycosylation sites in the linker between the catalytic domain and the carbohydrate‐binding domain,^15^ which may have significant effects on interactions with crystalline substrates.

The methods developed here can give information about binding and catalysis with relevant substrates, but questions still remain about processivity, endo versus exo preference, and potential clustering and cooperative behavior between multiple enzymes. One avenue to more fully characterize these aspects of catalysis will be single‐molecule measurements of kinetics. Recently, significant progress has been made in measuring chitinase activities by single‐molecule microscopy,^9^, ^30^, ^31^ and applying this approach to mammalian chitinases, ideally with native glycosylation, may help to break down the effects of different mutations on activity, give new insights into the function of the carbohydrate‐binding domain, and help to differentiate the enzymatic role of chitotriosidase and acidic mammalian chitinase.

## 4 EXPERIMENTAL PROCEDURES

### 4.1 *Protein preparation*

Constructs expressing a fusion of a protein A secretion sequence targeting periplasmic expression, AMCase or chitotriosidase, and a C‐terminal V5‐6xHIS as previously described^20^ were ordered from Atum (Newark, CA). Mutants of AMCase were generated via PCR mutagenesis. Plasmids were transformed into BL21 cells and expressed overnight in ZY Autoinduction media at 37°C for 3 hr followed by 19°C overnight. We added protease inhibitor at the temperature change to minimize proteolysis of periplasmically expressed protein. Pelleted cells were lysed via osmotic shock in a two‐step procedure. First, cells were resuspended in 20% Sucrose w/v, 20 mM Tris pH 6.5, 1 mg/mL lysozyme, 1 μL universal nuclease, with a protease inhibitor tablet. The resuspended cells were incubated at 37°C for 1 hr, and then pelleted via centrifugation at 15000 × g for 15 min. The supernatant was collected, and the pellet was resuspended in a wash buffer of 20 mM Tris pH 6.5 and 150 mM NaCl and incubated for 15 min at 4°C. The cells were centrifuged at 15000 × g for 15 min, and the supernatant was combined with the supernatant from the first step to form the combined lysate. The combined lysate was bound to a HisTrap FF column, washed with 100 mM Tris pH 6.5, 150 mM NaCl, and then eluted with a gradient into 100 mM Tris pH 6.5, 150 mM NaCl, 500 mM imidazole. Fractions were selected for further purification based on activity assay with a commercial fluorogenic substrate (described below). Active fractions were pooled and subject to dialysis overnight into 100 mM Sodium Acetate pH 4.5, 150 mM NaCl, 5% glycerol w/v followed by filtration to remove insoluble aggregate and dialysis into 100 mM Tris pH 6.5, 150 mM NaCl, 5% glycerol w/v. The protein solution was concentrated and separated via size‐exclusion chromatography on a Superdex S75 16/600. Fractions were selected based on purity as assessed via SDS‐page gel electrophoresis, and based on activity as assayed with a commercial fluorogenic substrate.

### 4.2 *Analysis of kinetic data*

Kinetic measurements were made in a range of substrate concentrations outside of pseudo‐first‐order conditions. To robustly measure rates of catalysis, we fit our data using non‐linear least‐squares curve fitting to simple relaxation models for enzyme kinetics:

$$A\left(1-e^{-k_{1}t}\right)+B$$

where A shows the asymptotic signal from the clearance of substrate, *k*_1_ is the rate constant of relaxation, and *B* is the background signal of the assay condition. To this end, we developed a small python library for relaxation modeling, which is available on GitHub: https://github.com/fraser-lab/relax. Generally a single‐step relaxation model was required, but in cases where residuals showed significant structure, additional steps were added as either relaxation or linear fits (in cases where kinetics were pseudo‐first‐order). Specific data analysis scripts using relax.py are available at https://github.com/fraser-lab/chitin_analysis.

### 4.3 *Continuous fluorescence measurements to quantify activity using commercial oligomeric substrates*

Catalytic activity was assayed using 4‐methylumbelliferyl chitobioside and 4‐methylumbelliferyl chitotrioside as described previously^32^ with one critical modification. 10 nM chitinase enzyme was incubated with varying concentrations of 4MU‐chitobioside or 4MU‐chitotrioside up to 433 μM in McIlvaine Buffer^33^ pH 7.0 at 37°C. The 4‐methylumbelliferone (4MU) fluorophore is quenched by a ß‐glycosidic linkage to a short chitin oligomer, which is cleaved by a chitinase enzyme, which generates fluorescence with peak excitation at 360 nm and emission at 450 nm. Previously, the reaction was quenched, and the pH was raised to maximize the quantum yield of the 4MU substrate. To avoid noise introduced by quenching and substrate concentration, we measured fluorescence at regular intervals during the course of the reaction without a pH shift and determined the rate using a single step relaxation model. This allowed us to measure rates of catalysis under a large range of conditions without needing to account for the proper time to quench to maximize signal without the reaction reaching completion. The processing for data collected from this assay is illustrated in Figure S1.

### 4.4 *Bulk clearance activity assay*

Borohydride‐reduced colloidal chitin was purchased as a powder from Megazyme (Bray, Ireland) and resuspended to 4% w/v in pH 7.0 McIlvaine buffer. Higher concentrations did not stay in suspension effectively. To remove soluble oligomers, the suspension was pelleted by centrifugation at 3200 × g, the supernatant was discarded, and the pellet was resuspended in McIlvaine buffer. This wash step was repeated a total of five times. A concentration series was prepared by serial dilution of this washed 4% w/v stock in McIlvaine buffer, and 50 μL of each substrate concentration was incubated with 50 μL of 200 nM chitinase at 37°C in a clear‐bottomed 96‐well microplate with a lid that was sealed around the sides with parafilm to minimize evaporation. Clearance of substrate was monitored by reduction of scattering at OD_680_ for 72 hr with shaking between reads to maintain substrate suspension. The processing for data collected from this assay is illustrated in Figure S2.

### 4.5 *Potassium ferricyanide reduction assay*

Four percentage w/v colloidal chitin was washed as above, and then diluted serially to generate a concentration range from was incubated with 1–100 nM chitinase for up to 18 hr at 37°C. At the endpoint of incubation, 50 μL of reaction mixture was quenched by the addition of 100 μL of 400 mM sodium carbonate. The insoluble chitin was pelleted by centrifugation at 4000 × g, and then 100 μL of supernatant was mixed with 100 μL of 0.6 g/mL potassium ferricyanide in a 96‐well microplate with clear bottoms and a lid that was sealed around the sides with parafilm to minimize evaporation. The microplate was incubated for 4 hr at 42°C to maximize the rate of the nonenzymatic reduction of potassium ferricyanide by solubilized reducing sugars. During incubation absorbance at 420 nm was read out in 1 min intervals. We found that progress curve analysis gave poor results for this data, and instead ultimately found the difference between the maximum and minimum absorbance to be a more robust measure of total reducing sugar generation in the 18‐hr incubation with chitinase. The processing of the data for this assay to generate rates is illustrated in Figure S3.

### 4.6 *Chitooligosaccharide oxidase coupled peroxidase assay*

Processing of colloidal chitin and resultant generation of new reducing sugar moieties was monitored, as previously described,^12^ by oxidation by chitooligosaccharide oxidase (ChitO), producing as a byproduct peroxide, which in turn is converted into a fluorescent signal by horseradish peroxidase (HRP) and QuantaRed peroxidase substrate.^34^ ChitO was purchased from Gecco Biotech (Groningen, the Netherlands), HRP and QuantaRed substrate were purchased from Sigma (St Louis, MO). Incorporating a fluorogenic HRP substrate improves the dynamic range of the experiment and enables real‐time observation of reducing sugar cleavage in a one‐pot reaction incorporating insoluble chitin, chitinase, chitO, HRP, and QuantaRed substrate. Briefly, a 50 μL solution containing 1–10 nM chitinase, 20 U/mL HRP, 100 nM ChitO, 0.5 μL of QuantaRed substrate, and 10 μL of QuantaRed enhancer solution in McIlvaine buffer pH 7.0 was mixed with 50 μL of washed colloidal chitin substrate, as prepared above, in a black 96‐well microplate with a lid to minimize evaporation. The plate was incubated with at 37°C and the fluorescence of the QuantaRed substrate was measured at 1‐min intervals for 16 hr. The progression of fluorescence over time was modeled as a relaxation process as described above, after subtracting the signal from a chitinase‐free control, which had signal that was modulated by the washing of the colloidal chitin. This enzyme‐coupled reaction is sensitive to reaction conditions, with artifacts introduced by insufficient excess of chitO or HRP as well as by insufficiently washed colloidal chitin. With careful washing of the colloidal chitin and sufficient prewarming of both enzyme and substrate solutions, rates can be reliably measured for chitin concentrations ranging from 0.0005% to 2% colloidal chitin w/v, and for chitinase concentrations as low as 50 pM. The processing of data from this experiment is illustrated in Figure S4.

### 4.7 *Random mutagenesis and screening*

Random mutations were generated using the commercial Genemorph II random mutagenesis kit (Agilent, Santa Clara, CA). The catalytic domain of acidic mammalian chitinase was amplified via error‐prone PCR with varying amounts of parent plasmid present. We titrated the amount of parent plasmid until each clone carried 1–2 mutations. We then performed restriction digestion using StyI and Eco130I and ligation using Quick Ligase to generate plasmids containing our mutations. We transformed these into electrocompetent BL21(DE3) *E. coli*. Individual colonies were picked and grown overnight in 96‐well deep‐well blocks, and then 20 μL of starter media was used to inoculate 300 μL of ZY media in deep well blocks, which was then used to express the protein at 30°C overnight. After expression, 50 μL of media from individual wells was mixed with 50 μL of 21.6 μM 4MU‐chitobioside in McIlvaine buffer pH 7.0, which had been prewarmed to 37°C. The mixture was monitored by fluorescence as described above, and compared to positive and negative controls, which had been expressed in the same plate. Mutants with increased activity were grown out, mini‐prepped, sequenced, retransformed, and expressed and rescreened in this manner in triplicate to confirm improved activity. Winners at this point were stored individually and pooled for further error‐prone PCR and screening.

## CONFLICT OF INTERESTS

BAB and JSF are inventors on a provisional patent application for the mutants described herein and their use in treating fibrotic lung disease. SJVD and RML are inventors on a pending patent application on the use of chitinases for treating fibrotic lung disease.

## Supporting information


**Figure S1** | Processing 4MU assay data
(a) Standards of 4MU were measured by fluorescence at 360 nm excitation and 420 nm emission and concentrations below 50 μM fit well to a linear regression (b) Progress curves of a concentration series of 4MU‐chitobioside were fit by a non‐linear relaxation analysis to extract initial rates. (c) Initial rates were plotted against substrate concentration and were fitted via non‐linear regression to a Michaelis–Menten curve to extract rate constants.
**Figure S2** | Data processing for colloidal chitin clearance assay.
(a) Concentrations from enzyme‐free controls were matched to absorbance, and for concentrations below 0.5% w/v a linear regression fit the data reasonably well. (b) Progress curves of a concentration series of bulk chitin were subtracted from their initial state, then fit by a non‐linear relaxation analysis to extract initial rates. (c) Initial rates were plotted against substrate concentration and were fitted via non‐linear regression to a Michaelis–Menten curve to extract rate constants.
**Figure S3** | Data processing for ferricyanide reduction assay.
(a) Concentrations from chitobioside controls were matched to absorbance, and for concentrations below 250 μM a linear regression was fit the data. (b) From progress curves for the non‐enzymatic reaction with potassium ferricyanide, the maximum and minimum values were subtracted from each other and scaled by the incubation time to extract the rate of generation of soluble reducing sugars. (c) Rates were plotted against substrate concentration and were fitted via non‐linear regression to a Michaelis–Menten curve to extract rate constants.
**Figure S4** | Data processing for chitO assay.
(a) Concentrations from chitobioside controls were matched to fluorescence after incubation with chitO, horseradish peroxidase, and quantared, and for concentrations below 30 μM a linear regression was fit the data. (b) From progress curves, a non‐linear regression was used to fit relaxation parameters to extract initial rates for a concentration series of colloidal chitin (c) Rates were plotted against substrate concentration and were fitted via non‐linear regression to a Michaelis–Menten curve to extract rate constants.
