# Supplementary material for: Differences in the chitinolytic activity of mammalian chitinases on soluble and insoluble substrates
Source: Protein Sci. 2020 Jan 21;29(4):966–77. doi: 10.1002/pro.3822 (PMC7096708; doi:10.1002/pro.3822)
Supplement: Supplementary file 1 — Figure S1 | Processing 4MU assay data (a) Standards of 4MU were measured by fluorescence at 360 nm excitation and 420 nm emission and concentrations below 50 μM fit well to a linear regression (b) Progress curves of a concentration series of 4MU‐chitobioside were fit by a non‐linear relaxation analysis to extract initial rates. (c) Initial rates were plotted against substrate concentration and were fitted via non‐linear regression to a Michaelis–Menten curve to extract rate constants. Figure S2 | Data processing for colloidal chitin clearance assay. (a) Concentrations from enzyme‐free controls were matched to absorbance, and for concentrations below 0.5% w/v a linear regression fit the data reasonably well. (b) Progress curves of a concentration series of bulk chitin were subtracted from their initial state, then fit by a non‐linear relaxation analysis to extract initial rates. (c) Initial rates were plotted against substrate concentration and were fitted via non‐linear regression to a Michaelis–Menten curve to extract rate constants. Figure S3 | Data processing for ferricyanide reduction assay. (a) Concentrations from chitobioside controls were matched to absorbance, and for concentrations below 250 μM a linear regression was fit the data. (b) From progress curves for the non‐enzymatic reaction with potassium ferricyanide, the maximum and minimum values were subtracted from each other and scaled by the incubation time to extract the rate of generation of soluble reducing sugars. (c) Rates were plotted against substrate concentration and were fitted via non‐linear regression to a Michaelis–Menten curve to extract rate constants. Figure S4 | Data processing for chitO assay. (a) Concentrations from chitobioside controls were matched to fluorescence after incubation with chitO, horseradish peroxidase, and quantared, and for concentrations below 30 μM a linear regression was fit the data. (b) From progress curves, a non‐linear regression was used to fit relaxation param [file PRO-29-966-s001.docx]

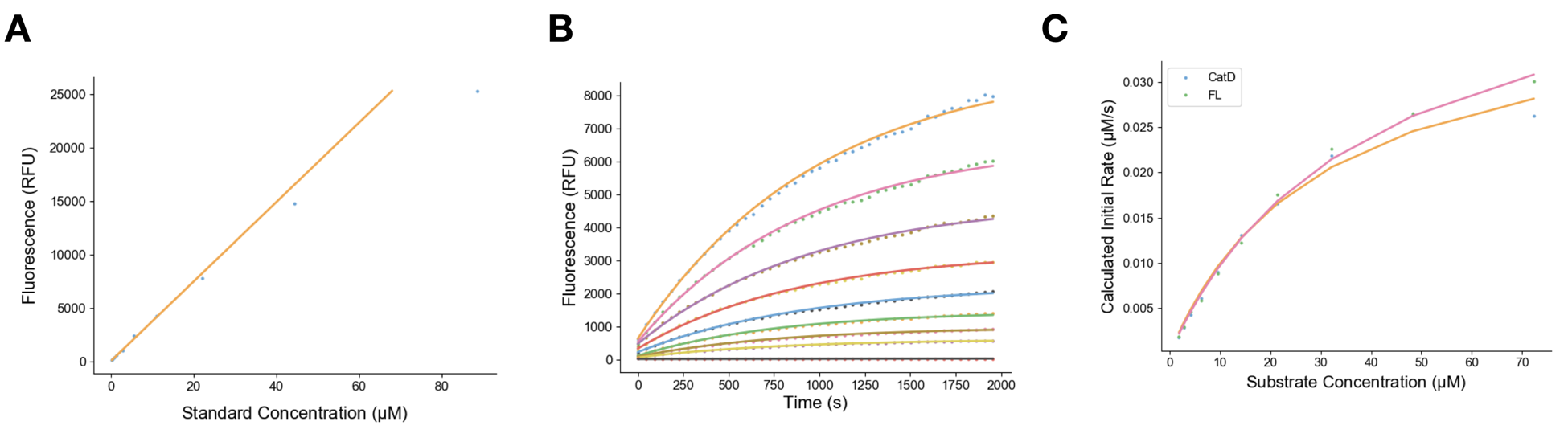


**Figure S1** | Processing 4MU assay data

(a) Standards of 4MU were measured by fluorescence at 360 nm excitation and 420 nm emission and concentrations below 50 µM fit well to a linear regression (b) Progress curves of a concentration series of 4MU-chitobioside were fit by a non-linear relaxation analysis to extract initial rates. (c) Initial rates were plotted against substrate concentration and were fitted via non-linear regression to a Michaelis-Menten curve to extract rate constants.


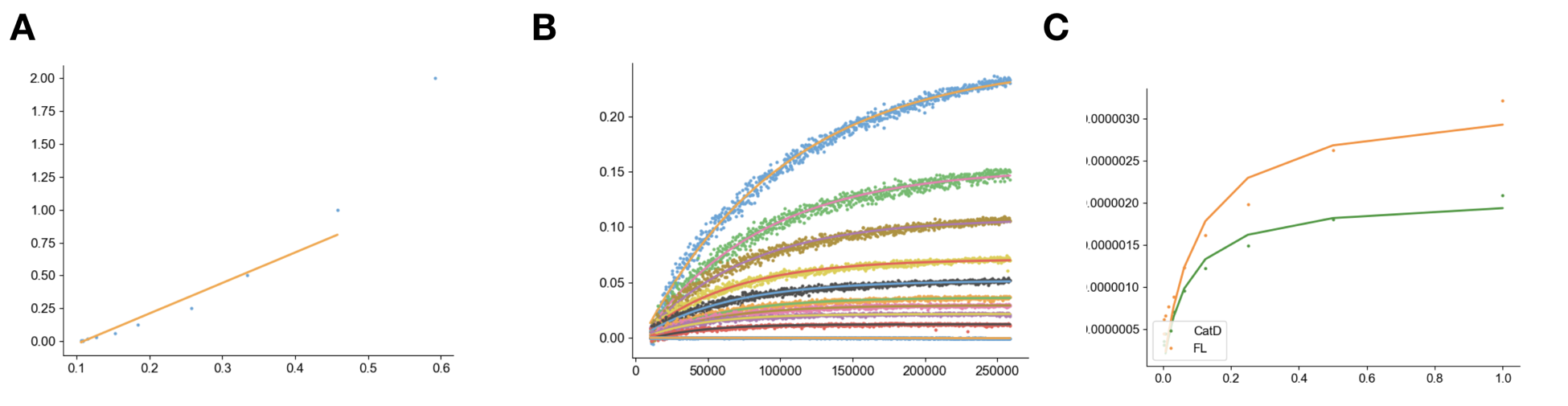


**Figure S2** | Data processing for colloidal chitin clearance assay.

(a) Concentrations from enzyme-free controls were matched to absorbance, and for concentrations below 0.5% w/v a linear regression fit the data reasonably well. (b) Progress curves of a concentration series of bulk chitin were subtracted from their initial state, then fit by a non-linear relaxation analysis to extract initial rates. (c) Initial rates were plotted against substrate concentration and were fitted via non-linear regression to a Michaelis-Menten curve to extract rate constants.


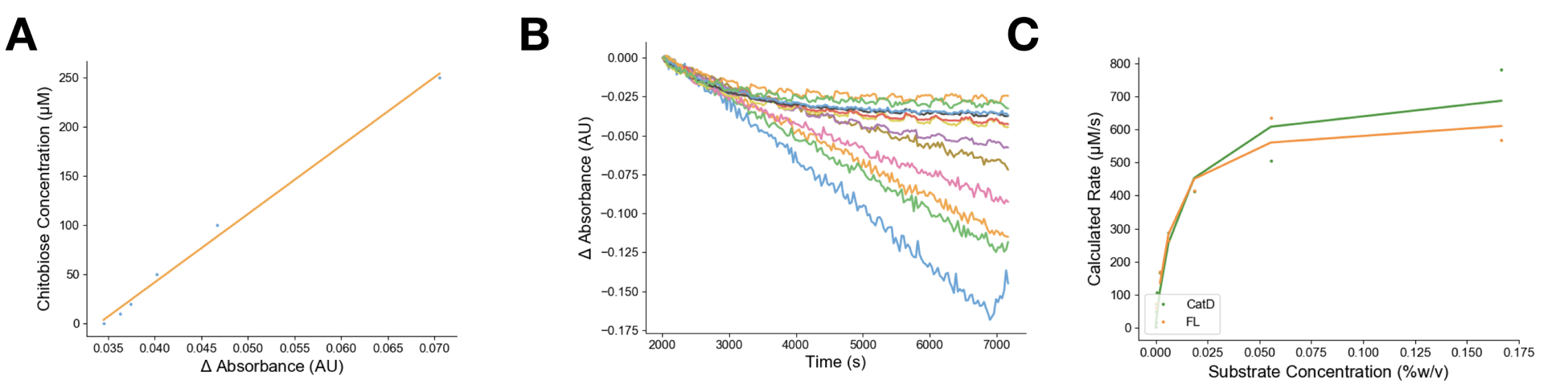


**Figure S3** | Data processing for ferricyanide reduction assay.

(a) Concentrations from chitobioside controls were matched to absorbance, and for concentrations below 250 µM a linear regression was fit the data. (b) From progress curves for the non-enzymatic reaction with potassium ferricyanide, the maximum and minimum values were subtracted from each other and scaled by the incubation time to extract the rate of generation of soluble reducing sugars. (c) Rates were plotted against substrate concentration and were fitted via non-linear regression to a Michaelis-Menten curve to extract rate constants.


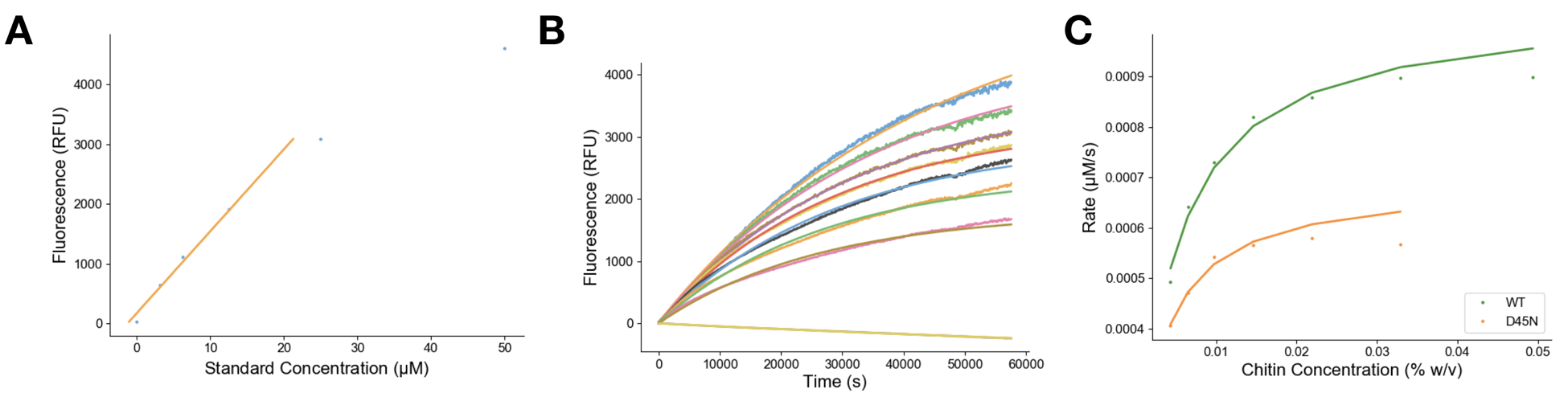


**Figure S4** | Data processing for chitO assay.

(a) Concentrations from chitobioside controls were matched to fluorescence after incubation with chitO, horseradish peroxidase, and quantared, and for concentrations below 30 µM a linear regression was fit the data. (b) From progress curves, a non-linear regression was used to fit relaxation parameters to extract initial rates for a concentration series of colloidal chitin (c) Rates were plotted against substrate concentration and were fitted via non-linear regression to a Michaelis-Menten curve to extract rate constants.
